# American Medical Association—Thirty-first Annual Meeting

**Published:** 1880-07

**Authors:** 


### Society Reports.

---

### ARTICLE VII.

### AMERICAN MEDICAL ASSOCIATION.—THIRTY-FIRST ANNUAL MEETING, HELD IN THE CITY OF NEW YORK, JUNE 1, 2, 3, AND 4, 1880.

The American Medical Association has held its thirty-first annual meeting, and has adjourned, leaving the record of a very pleasant social event and a fairly successful scientific gathering. The number of delegates was unprecedentedly large. By the evening of the second day 1,152 had registered, and the total number of delegates and visitors together was probably over 1,500. The country was well represented in all sections. The number of foreign visitors, however, was extremely small. Mr. Joseph Hutchinson, of Liverpool, Mr. Jonathan Hutchinson, of London, Dr. Ercolani, of Bologna, and Dr. Dillon, of Dublin, were the only persons from across the water whose names I saw mentioned. There were several medical gentlemen from Canada and South America, however. More than a hundred persons were made members by invitation.

THE ARRANGEMENTS

for the work and entertainment of the guests were well made, and everything went off smoothly, except the registering of delegates. It took nearly two days for this to be completed, and there was much inconvenience and delay occasioned. The pro- programmes of the work were published daily, instead of one for the four days, as heretofore. This did not suit the Association evidently, for it passed a resolution to have a single programme for the general sessions of the next meeting. A novelty in the way of medical jurnalism was the publication of a daily edition of *The Medical Record* containing a full account of the previous day's proceedings. This display of journalistic enterprise was commended by a vote of thanks from the Association to Dr. G. F. Shrady, the editor of *The Record*.

The meetings were held in the large hall and other rooms of the Young Men's Christian Association, and in the amphitheater of the College of Physicians and Surgeons, which is close by. The work was organized at first, as usual, in one general session and five special sections. In addition, however, on the second day, a temporary section on diseases of children was created, and by a vote of the Association this section was made permanent and was regularly officered for the next meeting.

THE SOCIAL PART

of the programme was a conspicuous one, and likely to be remembered as extremely pleasant. New York is the metropolis of our country, and some pride was felt that the entertainment of her guests should be worthy of the city. On the evening of the first day a reception was given at the Academy of Music. Music by Graffula and a supper by Delmonico were the special attractions. It seemed as though everybody went and took their friends. Two immense halls were crowded, and when the supper was served, were especially lively. The amount of salads, strawberries, ices, cakes, confectionery, sandwiches, lemonade, and iced coffee which was made to pass through peptonizing and endosmotic processes must have made the heart of the caterer quail. But there was enough, be it further recorded to the credit of the committee of arrangements and Mr. Delmonico. A large number of prominent medical men were on the floor, and a few of the laity watched the scene with interest. There were comparatively few ladies and but half a dozen grand toilets.

The next evening was taken up with an entertaiment at Booth's theater, given by Messrs. Reed and Carnicks, Scott and Bowne, the New York Pharmacal Association, and others. Mr. Booth acted Iago in Othello. The cast was an excellent one, and the play was doubtless much enjoyed by the many who rarely have an opportunity to see our greatest tragedian in one of his best characters. Thursday evening was given up to receptions by several private citizens and by the Mayor of the city. Mr. August Belmont received in the art gallery which forms part of his house. His collection of paintings is one of the best in the city, and his kindness in opening it to the medical profession was doubtless duly appreciated. The rooms were crowded the whole evening. Drs. Fordyce Barker and T. Gaillard Thomas received in the Academy of Medicine. On Friday afternoon, after the final adjournment of the meeting, there was a steamboat excursion up the Hudson, up the East river, and then down to Coney Island, where a lunch was served. This gave the guests an opportunity to see all the notable sights about New York. The excursion was given by Messrs. Wm. Wood & Co., to whom a vote of thanks was tendered during the trip.

THE BUSINESS DONE

by the Association was not very great, but all the questions that could well be disposed of were attended to. The Committee on Prize Essays announced that only one paper had been handed in and that it did not deserve a prize, so that none was given. As has been mentioned, a Section on Diseases of Children was created.

Considerable discussion was aroused by an attempt to exclude the naval delegation from the floor. An unsigned protest against its admission was sent to the Judicial Council, it being alleged that, a quack remedy, Holman's Liver Pad, had been bought and used by the Medical Department. It was voted to admit the delegation. Considerable discussion took place over a suggestion made by the President to substitute a periodical medical journal in place of the transactions, after the manner of the British Medical Association. The committee to whom the matter was referred, reported that it was better to wait the law of growth, and advised no immediate action in the matter. A committee of five will further examine into the matter and report at the next meeting.

The report of the Committee on Ozone consisted of a letter from Dr. N. S. Davis, its chairman, saying that it had been as yet, impossible to obtain satisfactory and reliable data upon the subject. He asked for $200 to continue the investigation, and it was given.

The constitution was so amended as to allow one delegate from the United States Marine Hospital Service.

The subject of the Metric system was elaborately reported on and discussed. Many hard knocks were given it, but the Association stood by its position of last year, and passed the following resolutions:

*First.* That it recommends the teaching and practice of the Metric system in medical colleges, clinics, dispensaries, etc.

*Second.* That it charges its Executive Metric Committee with the duty to report annually on the above institutions which teach, and those that do not teach the Metric system.

*Third.* That it authorizes said committee to enter into communication with the Metric Committee of the British Medical Association, in order to concert such plans as may render the use of the Metric system simultaneous and uniform in both countries.

Dr. Theophilus Parvin, chairman; Dr. Edouard Seguin, secretary; Drs. Edward Wigglesworth and F. R. Weisse, Executive Metric Committee.

The report of the Librarian showed that there were 3,258 volumes in the library; $200 was voted for continuing the librarian's work.

The following gentlemen were appointed delegates to European Medical Societies: Drs. Beverly Cole, Cal.; Benj. Lee, Philadelphia; M. A. Pallen, New York; and L. D. Bulkley, New York.

The committee appointed to consider the project of establishing a medical journal consists of Drs. W. W. Dawson, O.; J. R. Bronson, Mass.; W. H. Pancoast, Pa.; N. C. Husted, New York; J. S. Green, N. J.

The delegates to the Canadian Medical Association are: Drs. C. N. Brush, Buffalo; James R. Leaming, New York; D. H. Goodwillie, New York; Wm. Brodie, Detroit; W. B. Ulrich, Pittsburg.

A committee was also appointed to try and secure the official position for the members of the medical staff of the United States Navy, which is their due.

The subject of the Abuses of Medical Charities was referred to a committee consisting of Drs. Benj. Lee, H. G. Piffard, S. W. Gross, and J. W. Green.

The Treasurer's report showed the receipt of $5,025, and a balance in the treasury of $579.50.

An honorarium of $1,000 was granted the Permanant Secretary.

The following resolutions, sent up from the Section on State Medicine, were adopted:

*First.* Indorsing the National Board of Health.

*Second.* Recommending medical schools to establish a chair of State Medicine as part of the regular curriculum.

*Third.* That the name of the section shall be "Section on State Medicine."

*Fourth.* That the Committee on Prize Essays shall be Drs. S. E. Chaillé, La.; J. L. Cabell, Va.; and A. N. Bell, New York.

The following officers were elected for ensuing year:

*For President*—John T. Hodgen, M.D., of St. Louis, Mo.

*For Vice-Presidents: First*—W. H. Anderson, M.D., of Mobile, Ala. *Second*—Levi G. Hill, of New Hampshire. *Third*—Henry T. Holton, of Vermont. *Fourth*—H. Carpenter, of Oregon.

*For Permanent Secretary*—W. B. Atkinson, M.D., of Philadelphia, Pa.

*For Secretary*—Dr. W. H. Bradford, of Boston.

*For Assistant Secretary*—Dr. J. G. Cabell, of Virginia.

*For Committee of Publication*—The same as last year: Drs. W. B. Atkinson, T. M. Drysdale, Wm. Lee, R. J. Dunglison, Albert Fricke, S. D. Gross, and Casper Wistar, all of Philadelphia.

*For Chairman of the Section on Diseases of Children*—Dr. S. Jacobi, of New York.

*For Treasurer*—R. Dunglison, M.D., of Philadelphia, Pa.

*For Librarian*—William Lee, M.D., Washington, D. C.

*For Chairman* of the Section on Practice of Medicine, Materia Medica and Physiology—Dr. Wm. Pepper, of Philadelphia.

*For Secretary*—Dr. T. A. Ashby, of Maryland.

*For Chairman* of the Section on Surgery and Anatomy—Dr. H. McGuire, of Richmond, Va.

*For Secretary*—Dr. D. A. Eve, of Tennessee.

*For Chairman* of the Section on Obstetrics and Diseases of Women—Dr. James R. Chadwick, of Boston, Mass.

*For Secretary*—Dr. J. Taber Johnson, of Washington, D. C.

*For Chairman* of the Section on Medical Jurisprudence and State Medicine—Dr. J. T. Reeve, of Wisconsin.

*For Secretary*—Dr. R. G. Young, of Arkansas.

*For Chairman* of the Section on Ophthalmology, Otology, and Laryngology—Dr. D. S. Reynolds, of Kentucky.

*For Secretary*—Dr. S. M. Burnett, of Washington, D. C.

*For Members of the Judicial Council*, to fill vacancies—Drs. J. K. Bartlett, of Wisconsin; F. Staples, of Minnesota; D. R. Wallace, of Texas; J. S. Billings, of U. S. Army; J. H. Warren, of Massachusetts; and A. T. Woodward, of Vermont.

The Committee recommended that the next meeting of the Association be held in the City of Richmond, Va., on the *first Tuesday* in May, 1881.

*As Chairman of Committee on Arrangements*—Dr. F. D. Cunningham, of Richmond, Va.

During the course of the sessions a resolution of condolence was voted to the President for his recent great bereavement in the death of his son. The usual resolutions of thanks to the various persons who had contributed to the success and enjoyment of the meeting were also passed.

THE ADDRESS

by the President and the Chairman of the various sections formed prominent features of the general sessions. The opening address of welcome, by Dr. T. Gaillard Thomas, was a particularly eloquent and appropriate one. He referred to the changes that had passed over the country since the Association met in New York, sixteen years ago, and spoke of the wealth which New York had lavished upon its hospitals and charities. In conclusion he said that the end and object that brought the Association together was to emit and absorb thought, to give one another friendly greeting, and the hand-shake, and the kindly interchange of expression, and to advance the interests of a profession which most closely allied man to his Maker. Let it be hoped that the work done in the Association would lead those who tread in our steps to say, they met those who, like Ben-Adhem, loved their fellow-men, and strove earnestly in their cause. "In the name of the united profession of the city of New York and with outstretched hand and a glowing heart, I bid you welcome, thrice welcome to our home."

THE PRESIDENT'S ADDRESS

was most interesting in those parts where he took up practical points and discussed them. He began by congratulating the Association upon the marked improvement in numbers, work and kindly feeling among its members. He then went over some of the contributions to surgery and medicine which America had made, and showed that the reproach of having done nothing in this department is no longer deserved. The merits of the Metric system were thoroughly discussed.

A letter from Dr. J. B. Hamilton, Surgeon-General of the U. S. Marine Hospital Service, was read. This gave unqualified testimony to the value of and practical workings of the new system. Dr. Hamilton stated specifically that (1.) No serious difficulty had attended the adoption and exclusive use of the Metric system. (2.) That no mistakes had occurred through the usage. (3.) That volumetric methods were retained. He suggested that volumetric methods should be retained wherever the system was adopted.

The subject of the publication of the Transactions needed consideration, as there was great dissatisfaction with the present method. The plan of establishing a weekly periodical similar to the *British Medical Journal*, was broached. The history of the growth of the British Medical Association, and its rapid increase in prosperity after the establishment of a large journal, was given. Dr. Sayre was inclined to look favorably on the American Association undertaking a similar journal. In case this were done, the importance of having it well done and of having a competent man as editor, was insisted on.

The addresses by the

CHAIRMEN OF THE SPECIAL SECTIONS

Were all of considerable interest but were, none of them, remarkable performances. Neither did they cover the ground generally expected of them, that of noting the progress in the different branches of the sections upon which they were chairmen.

Dr. J. S. Lynch, of Baltimore, Chairman of the section on Practice of Medicine was not able to resist the general craze for discussing the importance of preventive medicine. He began by congratulating the association on the general good health of the country, a kind of congratulation that is apt to fall rather flat on an assemblage of doctors. The pathology of yellow fever was touched upon. The germs of this disease he said, were peculiar in the fact that they were not active when first emitted from the body, but required a certain time for development. Considerable evidence was given to support this statement. It being true, there was always time to destroy these germs when a case of yellow fever appeared, before they reached their stage of activity. Dr. Lynch referred to the possibilities of preventing or lessening scarlatina, diphtheria, consumption etc. He then went over the subject of antipyretics, and made an eloquent conclusion, describing the present position and triumphs of medicine.

The address of the chairman of the section on Surgery and Anatomy was devoted entirely to the subject of Preventive Trephining. After giving the history of trephining, he said that there were three different views held by surgeons on the subject: 1. That the trephine should never be used; 2. That it should be used simply to cure, *i. e.*, to relieve symptoms already developed; 3. That it should be used as a prophylactic to prevent the probable onset of symptoms. Dr. Briggs defended the third view, and urged the use of the trephine as a preventive. Dr. B. did not think trephining dangerous if done before the secondary effects of traumatism developed. In extensive comminuted fracture of the skull, the surgeon should not wait for symptoms of compression or inflammation to develop. The results of thus waiting and then operating are about as bad as of not operating at all. On the other hand, statistics show that prompt trephining for prevention of symptoms gives the best results. Of 106 cases, two-thirds had been saved by preventive trephining. In forty-two cases thus operated on by Dr. B., thirty-eight had recovered. He stated as essential to success, that there should be full antiseptic precautions, the use of the conical trephine, entire removal of all loose fragments of bone, and perfect drainage of the wound.

The address of the chairman of the section on State Medicine, Dr. James F. Hibbard, of Richmond, Indiana, was a very able and pertinent one. It has not however, so much of general interest, and I refer to it briefly. Dr. Hibbard stated, under the head of hygienic progress, that there had been published 1,525 documents on public hygiene during 1879, against 459 in all previous time; a statement that appears rather incredible. In discussing medical jurisprudence, Dr. Hibbard took ground against death by hanging, and mentioned the fact that Judge Heller, of Indianapolis, had for the first time, sentenced a man to be hung on Wednesday instead of Friday. The rest of the address was devoted to various problems is psychology and psychiatry.

THE WORK OF THE SECTIONS

began in the afternoon and lasted generally until five or six o'clock. The surgical section showed the most enthusiasm, and loquacity, twice keeping up its discussions until seven o'clock. Here as in other sections, there were a good many papers of practical value read and much instructive comment excited. Very few papers indeed, however, could take any high rank as showing elaborate study or rigid scientific method. One feature of the papers was that quite a number had been read or had appeared in substance before.

THE SECTION ON PRACTICE OF MEDICINE, MATERIA MEDICA AND PHYSIOLOGY

of which Dr. J. S. Lynch, Baltimore, was chairman, and Dr. W. C. Glasgow, of St. Louis, secretary, opened with a paper by Dr. Wm. H. Thomson, of New York, on

THE CLASSIFICATION OF MEDICINES.

Dr. Thomson is Professor of Materia Medica in the University College here, and he presented views on classification which he has for many years been teaching. He thought that all drugs may be assigned to one of two classes. The first-class includes those drugs which, whatever the dose, shows their special effect at once. Opium, emetics, cathartics, neurotics are examples of this class. They act immediately, producing symptoms; they affect functions rather than organs or structures. They show their effects equally upon sick and well. The other class includes those drugs whose action is not immediate, or if it is, it is not remedial. They produce their therapeutical results slowly. They do not produce symptoms, or if they do, they are not acting remedially. Iron, iodide of potassium, mercury and arsenic are examples of this class. They affect structures rather than functions; they produce their special effects on the sick only. The first of these two classes he called symptom-medicines because they were, as a rule, to be used in combatting symptoms and perverted functions only. The second class he called restoratives or disease-medicines, because they acted against the disease itself. It was laid down as a rule that when symptom-medicines were not given to the point of producing symptoms, they were not given in large enough doses. On the other hand, when disease-medicines were given in so large doses as to produce symptoms, the patient was getting too much. The symptom-medicines are those which are required in nearly all acute affections, as fevers, etc.; the disease-medicines were for chronic diseases.

The discussion on this paper was shared in by Drs. Roberts, Bartholow and Mary Putnam-Jacobi. Dr. Bartholow doubted the correctness of the classification and instanced cases where "symptom-medicines" did cure disease and where "disease-medicines" produced symptoms. Dr. Mary Putnam-Jacobi was the first woman who has spoken before the association. Her appearance excited much interest among the delegates, who crowded about her and listened with great attention. Dr. Putnam-Jacobi is a little woman with a pleasant face, a low voice and an entirely feminine manner. She disagreed with Dr. Thomson and made the point that the action of all drugs is molecular and hence is really structural; furthermore, that we already know what this molecular action is as far as some drugs are concerned, for example the alkaloids. It was impossible, therefore, to make any classification based upon the assumption that some remedies affect function, others structure.

Dr. M. O'Hara read a paper entitled "A Case of Occlusion of One or more of the Cerebral Sinuses." A young woman developed quite rapidly œdema of the right and then left side of the face, severe neuralgic pains, conjunctival ecchymosis, ocular protrusion and paralysis of the rignt sixth nerve. The case was carefully analyzed and the probability of thrombosis assured.

Rather more work was done upon the second day, the session opening with a paper by Dr. H. R. Hopkins, of Buffalo, N. Y., on

SPHYGMOGRAMS WITH NOTES OF AUTOPSIES.

The reader acknowledged the past deficiencies of the sphygmograph but predicted great things from its future careful study with perfected instruments. The idea at the basis of his paper was that a large number of chronic diseases have characteristic sphygmographic tracings, and by learning these the diagnosis could be helped. He showed a large number of sphygmograms, made by himself, illustrating the pulse in Bright's disease, locomotor ataxia, endarteritis, etc. A modification of Pond's sphygmograph was used. A large number of post mortem notes was read.

A paper of much practical interest was that of Dr. Robert W. Taylor, of New York, on the use of

CHRYSOPHANIC ACID IN THE TREATMENT OF SKIN DISEASES.

The author indorsed the drug as having as much specific effect in some cases as quinine. He recommended it in chronic or subacute skin affections where there is superficial infiltration and in scaly diseases, lichen, papular and scaling syphilides, some forms of eczema, indurated acne; but above all psoriasis is benefited by the remedy. The strength should be about gr. x to ℥j of simple ointment. The objections to the acid are its irritant and staining properties. It is not anti-pruritic.

One of the best papers read before the section was that by Dr. Wm. Pepper, entitled

A FURTHER CONTRIBUTION TO THE LOCAL TREATMENT OF PULMONARY CAVITIES.

Dr. Pepper referred to the all but total uselessness of inhalations and sprays in the treatment of pulmonary cavities. In his experience it was very rare indeed to see these heal up. Any method which might help this was to be regarded with favor, therefore. He had tried the direct injection of Lugol's solution (m. x, m. xv, or ʒj to ℥j) in seventeen cases; 291 injections were made in all. The results had been encouraging and he intended to continue the practice. In several post mortems on the cases, he found that the cavities had healed up. The patients all had confidence in the treatment. He had seen no ill effects and he believed it certainly demonstrated that the injections could be made with safety. The special indications for the operation were the presence of well defined cavities with not much osseous tissue about them.

In a rambling paper by Dr. J. R. Uhler of Baltimore "On Restorative Medicines" an ingenious method of

MEASURING UREA

was described. It consists in taking two bottles, one of which just fits into the other. The smaller one is attached by a wire to the cork of the larger. In the smaller is placed the urine; in the larger a mixture of liquor sodæ chlorinatæ and salt. The one is placed carefully in the other and both are weighed. They are then shaken up and the contents of the two bottles mixed together. Chemical decomposition takes place and the nitrogen of the urine is set free. This is allowed to pass off and the two bottles are again weighed. The difference in weights represents that of the nitrogen; from which can be estimated that of the urea.

The session of the third day was opened by Dr. A. D. Rockwell of New York with a paper on

THE ELECTRICAL TREATMENT OF EXOPHTHALMIC GOITRE.

Dr. R. said that the Faradic current was of some value in this disease when applied by the method of general Faradization. The main reliance, however, must be in the galvanic current. In giving this the cathode is placed over the cilio-spinal center and the anode in the auriculo-maxillary fossa; the anode, after a few minutes of stable treatment, is drawn along the inner border of the sterno-cleido muscle to its lower extremity. The current is then reversed and increased in strength. Of nine cases treated this way, four completely and three partially recovered.

A very practical paper was read by Dr. L. Duncan Bulkley of New York on the

USE OF SULPHUR AND ITS COMPOUNDS IN DISEASES OF THE SKIN.

Pure sulphur is given internally in few skin diseases, but is beneficial when there is eczema of the genitals and about the anus. Sulphide of calcium is of considerable value in the more pustular forms of acne, in hordeolum, furunculosis and suppurating buboes. The drug is liable to be poor. Dose, gr. $\frac{1}{4}$, q. i. d. Dr. Bulkley had no data which enabled him to indorse the natural sulphur waters. Externally, sulphur is most potent as a destroyer of parasites (*e. g.* animal and vegetable) as those of scabies, favus, ringworm and tinea versicolor. Pure sulphurous acid is the best form for these. Sulpur baths were probably of not much more value than ordinary local applications of sulphur. The discussion that followed this paper turned largely upon the sulphide of calcium. Some asserted that it interfered with digestion. On the whole the value of the drug was indorsed.

Dr. V. P. Gibney read a paper on the "Strong Galvanic Current in the Treatment of Sciatica." By giving the current as strong as the patient could bear it for ten minutes every day for a week or two, he had entirely relieved 24 out of 32 cases.

Dr. L. Turnbull read a paper on "Hydrobromic Ether." There was not time for hearing it all, however.

THE SECTION ON SURGERY AND ANATOMY

was opened with a paper on "Spinal Extension; its Modes Means and Motives," by Dr. Benj. Lee, of Philadelphia. It embraced ideas which he had to some extent given to the public before. He showed his surgical table, by which systematic extension can be made by the patient himself.

### PHIMOSIS AS A CAUSE OF NERVOUS SYMPTOMS

was the title of a paper read by Dr. Geo. M. Beard, of New York. There were, he said, a number of nervous symptoms which were either caused or kept up by phimosis and sometimes even by adherent and redundant prepuce. Some of these symptoms are: morbid fears, flushings, persisting flushings, sweating of the hands, palpitation, muscular twitchings, etc. Sometimes phimosis exists without producing symptoms until the system gets run down. Circumcision helps to relieve the condition, but immediate or startling results are not to be expected. Out of 80 cases of neurasthemia, 31 had phimosis or adherent and redundant prepuce.

In the discussion upon the paper additional cases were related showing the effect of phimosis on the nervous system. Dr. Hard of Illinois said that he had advised circumcision in cases of insanity. This had been sometimes done merely to make an impression on the patient and had been practiced where the prepuce was not redundant.

A paper was read by Dr. John T. Hodgen, of St. Louis,

### ON SECTION OF THE INFRA-ORBITAL AND INFERIOR DENTAL NERVE FOR NEURALGIA.

The speaker described a hook or elevator by which he drew out the nerve, and thus made the section further back. The full details were then given; but they have been published in part before, and need not be given here. Dr. H. had operated twenty-four times on twelve patients. All were greatly relieved; some were entirely cured. In the discussion that followed, Dr. Jas. R. Wood described his own method of operating in such cases. He cut through the walls of the antrum, and cut the nerve on the proximal side of Meckel's ganglion. That ganglion ought to be removed, he thought, in order to secure success. Even then, in the majority of cases, the disease would return.

Dr. Chas. T. Stillman, of Plainfield, N. J., read a paper on "Newly Devised Orthopedic Appliances, including the Sutor splint."

Dr. Pancoast, of Philadelphia, read by title a paper on

CERTAIN METHODS IN SURGERY AND CONSIDERATIONS OF THE ETIOLOGY AND PATHOLOGY OF WHITE SWELLING OR SYNOVITIS OF THE JOINTS, IN REGARD TO THE PRACTICE OF EXTENSION IN TREATMENT.

He then spoke on various surgical topics. He showed samples of black silk which he preferred for sutures, because the white silk commonly contained lead salts. He described his method of amputating at the metacarpo-phalangeal articulation. He always employed the volar flap, and his success was invariably good. He showed a urethrotome; in using this, he made shallow cuts and then dilated. Twenty-one cases had been successfully treated with the new instrument. In regard to joint affections, the two points insisted on were that cartilage did not become inflamed, but that the chief seat of the disease was the synovial membrane; that the essential point in treatment was to relieve the synovial membrane, rest and not extension, and to relieve the articular cartilages. Extension might even make matters worse. Along with rest the more efficient revulsion was the hot iron.

The discussion turned upon the puncture of joints. Dr. Martin spoke of its freedom from danger. He had made 240 exploratory articular punctures in which he had purposely abstained from antiseptic precautions and had never found the slightest symptom of inflammatory reaction.

In a paper by Dr. Jas. L. Little, of New York, read on the second day on "Compound Complicated Hare-lip," several successful cases were related. The intra-maxillary bone was removed in the two instances, with the best results. In the discussion, instances of operating for hare-lip four and eight hours after birth were cited and early operations commended.

CYSTOTOMY AND CYSTITIS IN THE MALE, was the title of an admirable paper by Dr. Robert F. Weir, of New York. The credit of originating the operation was given to Dr. Willard Parker, who announced it in 1867. Dr. Weir had collected forty-seven cases, of which thirteen died, ten deaths being due to advanced kidney disease; twenty-three were cured, seven were relieved, and four were not benefited. The operation consists in cutting into the bladder, preferably by the lateral incision, and in keeping the opening patent, at first with the finger daily inserted, afterwards with a tube. Dr. Weir advised removal, if possible, of enlargement of the prostate, if that existed.

Dr. Gouley, in discussing the operation, said that no patient would get well after cystotomy unless there had been a free division of the urethro-vesical orifice.

Dr. Turnbull read a paper on " Skin Grafting." During its discussion, Dr. Burchard mentioned a case in which 200 grafts had been taken from an amputated thigh three hours after its removal, and inserted into an immense ulcerated surface over the breast of a woman. A stimulus was imparted to the edge of the wound by this. Afterwards, 500 more grafts were put on the wound, with considerable benefit.

In a paper on the Treatment of Intestinal Obstruction, by Dr. W. A. Byrd, of Quincy, Ill., the following conclusions were reached: No one should be allowed to die from intestinal obstruction without at least an exploratory incision. If there is great tympanitis, the intestines should be aspirated. In cases where abdominal section is contra-indicated, an artificial anus should be formed.

THE PATHOLOGY AND TREATMENT OF SYPHILIS

was the title of a paper by Dr. F. N. Otis, read on the third day. Dr. Otis' views, as there given, have been announced before, although not as fully and exhaustively as at this time. He believes, essentially, that the virus of syphilis is not a physical entity, but an influence. This " influence " causes excessive local accumulations of leucocytes, which in turn cause the sclerosed lesions and symptoms of the constitutional disease. The object of treatment must be to get rid of this excessive cell accumulation. Small doses of mercury long continued helped this.

A paper, "On the Development of the Osseous Callus," of high scientific value, was read by Dr. H. C. Marcy, of Cambridge. He stated that there was no such thing as primary union of bone. The interest of the paper was increased by the exhibition of some beautiful microphotographs.

In a paper on " Lupus," by Dr. H. G. Piffard, of New York, the disease was classed among the scrofulous skin affections. He divided it into three forms : the non-ulcerative, the superficial ulcerative, and the deep ulcerative. He treated it with thorough excision and subsequent application of the actual cautery, or chloride of zinc.

A paper on "The treatment of Syphilis," by Dr. Chas. R. Drysdale, of London, Eng., contained no especially new ideas. The same may be said of some remarks by Dr. Martin on the value of his Elastic Bandage.

A paper having some novelty was that of Dr. H. T. Campbell, of Augusta, Ga., "On the Radical Cure of Inflammation by Ligation of the Vessels of Supply." The title of the paper shows the author's idea. He related seven cases of inflammations affecting the lower extremity and eight of the arm in which he had ligated vessels. The results were all satisfactory, sometimes surprisingly so.

Owing to lack of time five other papers were read by title only, and referred to the committee.

THE SECTION ON OBSTETRICS AND DISEASES OF WOMEN

was presided over by Dr. G. M. B. Maughs, of St. Louis, in the absence of the regular Chairman, Dr. A. H. Smith. The Secretary was Dr. Robt. Battey, of Rome, Ga.

The first paper was by Dr. J. Marion Sims, of New York, on "Battey's Operation in Epileptoid Affections." Dr. S. believed that Battey's operation would soon be recognized as a legitimate one. The speaker had performed it eleven times. He now reported the results of the last four. The first was on a married woman, aged 30. She had suffered many years from dysmenorrhœa, and of late years from epileptic attacks during menstruation. She had, when examined, vaginismus, vaginitis, retroversion, stenosis of the cervical canal, and both ovaries were enlarged and tender. After the operation she was perfectly well. The second case was one of hystero-epilepsy in an unmarried female of 31. She had suffered constantly for many years from pain radiating from the pelvic organs, and from dysmenorrhœa. She also had a retroverted uterus and tender ovaries. The operation relieved her from pain, but she is still weak. The third case was also one of hystero-epilepsy, and the patient died, from the bromide of ethyl, as Dr. Sims thinks; from the operation, as Dr. Turnbull thinks. The fourth case was that of an unmarried female, aged 21. She had had convulsions with occasional intermissions ever since the age of 10½. The aura started from the uterus and radiated trward the ovaries. She did not lose consciousness. The operation was performed last January, and did not secm to do much good.

Dr. Sims always performed the operation by making an abdominal incision. The uterus is meanwhile held up by an elevator thrust into its cavity and, the peritoneum being opened, the ovary is seized, its pedicle ligated and the organ removed.

In the discussion that followed, the opinion was developed that an operation through the vagina was safer, but sometimes when the ovary was bound down it could not be removed that way, hence success was not so certain.

Dr. M. A. Pallen read an essay on the same subject, but discussed it more exhaustively, giving the history, etc. Dr. P. had operated four times and conceived the operation independently of Dr. Battey, but a short time later than that gentleman. He had operated three times: for epileptiform dysmenorrhœa, hystero-epilepsy and for persistent catalepsy in the insane. The second case only recovered and was somewhat improved.

The second day's session was opened with a paper by Dr. Jos. Tabor Johnson, of Washington, on the

MANAGEMENT OF THE THIRD STAGE OF ABORTION, WITH RETENTION OF PLACENTA AND MEMBRANES.

The point made in the paper was that the patient ought not to be left until the uterus was completely emptied and firmly contracted. This point was indorsed by several gentlemen who followed in discussion. Dr. Morris, of Baltimore, "speaking for the men of the past generation," said that he thought the placenta should be allowed to remain so long as no injurious effect resulted. Two other gentlemen agreed with this speaker.

Dr. Isaac E. Taylor, of New York, read a paper on "Gastro-Hysterotomy; being Remarks on and Exhibition of a Full-term Uterus, Removed by Laparotomy." The patient had a deformed pelvis, and had been delivered by gastrotomy five years before. In this case Dr. Taylor had first delivered the child by Cæsarean section ; he had then passed two ligatures around the uterus, about an inch apart, between the cervix and the body of the uterus, where he believed there was a ductile isthmus. He then cut off the uterus between the two ligatures. The patient did well, except for an attack of phlegmasia dolens. She recovered from this ; but on the twenty-seventh day died of cardiac thrombosis. Dr. Taylor believed the death had nothing to do with the operation. He spoke strongly in favor of it. Out of fifty cases so far reported, twenty-one had recovered.

Dr. T. Gaillard Thomas, of New York, read a paper entitled, "Clinical Contributions to the Subject of Removal of the Uterus, in Whole or in Part, by the Extirpation of Tumors Connected with that Organ." There are three conditions, he said, which justify removal of the uterus: malignant disease ; as an addendum to Cæsarean section ; and in order to render practicable the removal of tumors which take their origin in its tissues, or which, arising in the ovaries, have attachments to the uterus too close to be separated. It was of the third class of conditions that he wished to speak. He related some illustrative cases. In one the fundus, in another the body, and in the rest the whole uterus was removed. Of the seven, four recovered. The three fatal cases were all those of large, solid fibroid tumors. Of the four successful cases, one was a large, solid fibroid, one a fibrocyst, and two were ovarian cysts, extending down into the broad ligament, and having a large amount of solid material in the walls. It was concluded that the fibro-cystic tumors whose size could be diminished by tapping, offered the best chance of success, since they allowed shorter abdominal incision.

On the third day the first paper was read by Dr. Addinell Hewson, of Philadelphia, on

THE TREATMENT OF FIBROIDS OF THE UTERUS BY DRY EARTH.

Dr. H. had been studying this subject for twelve years. He had treated fifty cases ; in all of them he had had satisfactory results ; in some complete cure. The material employed was a fine yellow clay, such as is used in making the best Philadelphia brick. This was first moistened, so that it would fit better; a layer a quarter of an inch thick was then placed around the abdomen and back, covered with a thin sheet of cotton batting, and secured by a many-tailed bandage. Almost immediate relief from pain was secured, tenderness disappeared, and great reduction in the size of the abdomen soon took place. In a case related, the size diminished one-half in three weeks. In a case that died, while under treatment, from an intercurrent affection, a large fibroma of the uterus was found undergoing cystic degeneration. Dr. Hewson thought that the action of the earth was a chemical one. Ordinary potter's clay would not do so well.

Dr. B. F. Dawson, of New York, read a "Clinical Report on a Modified Operation for Cystocele." In this operation he folds the redundant anterior vaginal wall into the bladder. The other steps were much like those of Schroeder's operation described in 1876.

Dr. Robert Battey, of Rome, Georgia, related a "Case of Still-birth, with Resuscitation After Two Hours and Five Minutes." Dr. Battey did not think a child should be regarded as dead because its heart had ceased to beat. In the present case, the child was wrapped in hot flannel, while he made inflation by the mouth.

Dr. Beverly Cole, of San Francisco, made some remarks on the preparation of sponge-tents. These called from Dr. M. A. Pallen a strong expression of disapproval of the use of sponge-tents.

THE SECTION ON OPHTHALMOLOGY, OTOLOGY AND LARYNGOLOGY

has for its chairman, Dr. Lawrence Turnbull, of Philadelphia; for secretary, Dr. Eugene Smith, of Detroit. There were several excellent papers read before this section. Dr. W. H. Daly, of Pittsburg, Pennsylvania, read a paper on "A Case of Syphilitic Stenosis of the Larynx, with Fibrous Adhesive Bands of the True Vocal Cords; Tracheotomy; Rupture of the Bands; and Cure of the Stenosis by General and Local Treatment." The title of the paper indicates the character and history of the case, which was not discussed. In a paper on the "Lesions of the Larynx in Pulmonary Phthisis," Dr. Carl Seiler, of Philadelphia, asserted that the ulcerative process begins in the glands and not in the mucous membrane. Dr. S. did not believe that the larynx should be considered a separate local habitat of the phthisical disease.

Dr. Knapp, of New York, read a paper on "Tumors of the Lachrymal Glands; their Pathology and Treatment, with Demonstrations." A number of cases were cited. He believed that all those tumors begin as adenomatous growths; unfrequently their character becomes mixed, a considerable part being myxomatous.

In a paper read the second day by Dr. W. H. Daly, "On the Therapeutical Value of the Galvano-Cautery in Diseases of the Naso-Pharynx," the opinion was given that this form of cautery is surer and less painful than other kinds, but is more expensive and capricious. It would not probably become popular.

A paper by Dr. S. J. Jones, of Chicago, "On the Introduction of liquids into the Eustachian Tube and Middle Ear," elicited considerable discussion. Dr. J. introduces slightly saline injections into the middle ear by means of a Eustachian catheter, and believed them of value in dry, chronic, non-suppurative inflammation. Drs. Knapp, Holcombe and Pomeroy, disagreed with the reader; Drs. Ritchey, Chisholm and Noyes, of Detroit, agreed with him as to the use of these injections.

On the third day Dr. B. Joy Jeffries spoke on "Color Blindness." He said he had examined 185 of the delegates and found five color-blind.

Dr. David Hunt, of Boston, read a paper on "The Variability of the Human Eye," and ventured an opinion that myopia often was the result of embryological changes brought about by the course of development. Dr. E. Gruening, of New York, exhibited a "Magnet for the Removal of Particles of Steel and Iron from the Interior of the Eye." Dr. T. R. Pooley, of New York, read a paper on "The Detection of the Presence and Location of Pieces of Steel and Iron in the Eye, by the Indicator of a Magnetic Needle." The author formulated the following conclusions: 1st. That a steel or iron body in the eye may be detected by a suspended magnet when the body lies near its surface. 2d. The presence and position of such a foreign body may most surely be found by making it a magnet by induction, and then testing for it by a minute suspended magnet. 3d. The intensity of deflection of the needle is proportionate to the depth of the body. 4th. Changes of deflection of the needle indicate changes of position in the foreign body. The instrument used is a magnetized needle suspended by a silk thread.

Dr. E. S. Peck, of New York, related an interesting case of "Primary Conjunctival Lupus."

THE SECTION ON STATE MEDICINE, as it is now called, had for chairman, Dr. Jas. F. Hibbard, of Richmond, Indiana; and for secretary, Dr. Thos. F. Wood, of Wilmington, North Carolina. The opening paper was by Dr. C. R. Drysdale, of London, England, on "Death-rate of the Rich and Poor." Dr. D. asserted that it was poor wages more than anything else which caused large death-rates among the poor. He also asserted that the death-rate among the poor should be lowered, and that it should be considered immoral for a poor man to have a large family. Papers were read by Dr. J. S. Billings, on "The National Board of Health," and by Dr. E. H. Parker, of Poughkeepsie, on "The Relations of the Medical and Legal Professions to Criminal Abortion." The latter advocated the full penalty of the law for abortionists, and urged that the prevalent idea that abortion is not a grave offense should be counteracted in every possible way. Dr. A. N. Bell read a paper on "Unsanitary Engineering," which caused considerable discussion.

On the second day a paper was read for R. C. Kedzie, of Lansing, Michigan, on "The Temperature of Living-Rooms;" and one by Dr. A. L. Carroll, of New Brighton, New York, on "The Personal Factor in the Etiology of Preventable Disease."

The third day was taken up to some extent with the discussion and passage of two resolution. The first, offered by Dr. J. S. Billings, was as follows:

"*Resolved*, That this Association approves of the plans proposed by the Superintendent of the Tenth Census for the collection of data with regard to the insane and idiots of the United States, and that it urges upon physicians that they should aid in this work, as far ar possible, by carefully replying to the schedule of queries on this subject which will be sent them from the Census office.

The second was an indorsement of the National Board of Health. "A Report on the Intervention of the Physician in Education," was read for Dr. D. R. J. O'Sullivan, of New York. It urged more attention to the sanitary condition of school-houses and the health of the pupils.

Dr. J. V. Quimby, of Jersey City, read a paper on "The Criminal Use of Chloroform." He stated among other things, that it had been shown by experiment that persons could be chloroformed while asleep without being awakened. Papers were read by Dr. W. H. Lathrop, of Tewkesbury, Massachusetts, on "Thoughts Regarding Alms Houses," by Dr. Antisell, of Washington, D. C.; on "Suspicions of Poisoning;" and by Dr. W. F. Thoms, of New York, "On Humane Societies."

THE SECTION ON DISEASES OF CHILDREN

was a new organization and no papers had been prepared especially for it. Very good work was done by those who took part however, and it had a fair-sized audience. It was organized with Dr. S. C. Busey, of Washington, D. C., in the chair, and Dr. Frank Woodbury, of Philadelphia, secretary. Dr. A. Jacobi, of New York, made an opening speech and advocated the claims of pediatric medicine. He made a distinction between the necessity of studying the specialities and practicing them. Alluding to the growth and encroachments of the various specialties he said: "The general practitioner will in future obtain, as the legitimate province of his practice, the male half of mankind, and very old women, and very young children, provided he will keep his hands off their eyes, ears, nervous system, lungs and heart, urinary organs, venereal diseases, nose, pharynx, larynx, skin, hair and corns." He pointed out the fact that the multiplication of specialists is due, first, to the immense progress of the science; and, secondly, to the attainment of special skill and dexterity by certain individuals, that leading them to select certain branches as their favorite practice."

Dr. S. C. Busey then read a paper on "Bright's Disease of Children Caused by Malaria. He related three cases in which children who had suffered some time from malarial poisoning, subsequently had Bright's disease. He did not insist positively upon their being a relation of cause and effect, but believed that the sequence should be kept in mind. Dr. Jas. L. Green reported a case of "Congenital Multiple Lymphectasia," in a male born at eight months. There was a large cystic tumor upon the posterior portion of the pelvis. On the abdominal region in front were two other smaller tumors. Two days later the fluctuating tumor was aspirated and 120 grams of fluid removed, which was clear, straw colored, specific gravity, 1007, slightly saline, and albuminous (about $\frac{1}{8}$) on boiling; nothing but a few blood corpuscles were observed under the microscope. In ten days the sac refilled to its former size. Dr. A. Jacobi now saw the case and pronounced it one of lymph angiectasis, and recommended removal of a small portion of the fluid, and injection of a small amount of iodine. On two subsequent occasions this operation was repeated, very little disturbance being produced; the slight uneasiness of the child passed away in two hours. This tumor has considerably diminished in size, but the tumors in front, upon which no treatment was attempted, have increased, and a small one has been developed upon the left hip opposite the acetabulum.

On the discussion, Dr. Jacobi said that since the tumor is now smaller than at the beginning, the tincture of iodine injections should be continued, and more frequently repeated, being careful, however, not to excite too much febrile reaction in the child. He distinguished two forms, one in connection with lymph trunks, and the other isolated. In the former the injection of iodine is the best treatment; the latter kind may be extirpated, and he had twice dissected out such isolated tumors in adults.

On the third day, Dr. A. Jacobi read the history of a case of "Congenital Atrophy of the Liver."

In speaking of the case, Dr. Jacobi also reported a case of syphilitic liver which had recovered—the only infant that he had known to survive the disease. The child is now a year old, and appears to be healthy.

In the treatment, where the evidence of syphilis appears early, a prompt mercurial impression is required, and he had followed

Lewin's plan of giving the bichloride hypodermically, with excellent results. The salt was dissolved in water and filtered before using. In other cases, where such a rapid effect is not needed, he had followed the usual plan of mercurial inunction.

Dr. Jacobi also reported

A CASE OF SUPRAPUBIC LITHOTOMY

in a child, six years of age, which had suffered with the symptoms of stone in the bladder for a year.

The operation was performed without difficulty, under chloroform, and a medium-sized phosphatic stone removed. This was followed by persistent suppuration, which was more than would be expected simply from a mucous membrane. The case had not yet entirely recovered.

In discussing the case, Dr. Jacobi referred to recent observations upon sub-mucous gastric abscess, and inquired whether a similar condition could not exist in the bladder. These accidents of peritonitis and extensive suppuration, as a consequence of epicystotomy, may prove to be a serious bar to a method of operation for lithotomy that otherwise has much to commend it. It may be that the preliminary distention of the bladder required, may have injurious results in a bladder which is contracted, and the subject of concentric hypertrophy and chronic irritation from the presence of the stone. If the bladder be much diseased, the forced injection may of itself produce serious consequences. In the case reported, no injury or irritation of the bladder was caused in operating, beyond the simple incision.

One or two other cases were also reported to the section and discussed.

---

THE Faculty of Rush Medical College have changed the time allotted to their practitioners' course, and have announced that the lectures for post-graduates will be given in the month of April next year, after the annual winter session is completed.

